# Aluminum-Magnesium Hydroxycarbonate/Azo Dye Hybrids as Novel Multifunctional Colorants for Elastomer Composites

**DOI:** 10.3390/polym11010043

**Published:** 2018-12-29

**Authors:** Bolesław Szadkowski, Anna Marzec, Przemysław Rybiński, Waldemar Maniukiewicz, Marian Zaborski

**Affiliations:** 1 Institute of Polymer and Dye Technology, Faculty of Chemistry, Lodz University of Technology, Stefanowskiego 12/16, 90-924 Lodz, Poland; boleslaw.szadkowski@edu.p.lodz.pl (B.S.); marian.zaborski@p.lodz.pl (M.Z.); 2 Department of Environmental Protection and Modeling, The Jan Kochanowski University, Świętokrzyska 15, 25-406 Kielce, Poland; przemyslaw.rybinski@ujk.edu.pl; 3 Institute of General and Ecological Chemistry, Lodz University of Technology, Zeromskiego 116, 90-924 Lodz, Poland; waldemar.maniukiewicz@p.lodz.pl

**Keywords:** aluminum-magnesium hydroxycarbonate, elastomer composites, hybrid pigments, barrier properties, flame retardancy

## Abstract

This study presents the preparation and characterization of new organic-inorganic pigments based on aluminum-magnesium hydroxycarbonate (LH) and azo dyes. Solvent resistance studies, XRD, SEM, and TGA confirmed the successful formation of hybrid pigments, which were characterized in terms of their physicochemical properties. The new hybrid pigments were applied in acrylonitrile-butadiene (NBR) and ethylene-propylene (EPM) rubber composites and cured with sulfur and peroxide curing systems, respectively. The mechanical properties, dispersion quality, and flame-retardant properties of the NBR/hybrid and EPM/hybrid pigment composites were determined by dynamic mechanical analysis (DMA), SEM, and microscale combustion calorimetry (MCC). Complex experimental investigations revealed that the layered nature of hybrid pigments could improve the barrier ability and flame retardancy of elastomer composites. In comparison to unmodified aluminum-magnesium hydroxycarbonate, the modified LH dye structures contributed to significantly decrease the heat release rate and the total heat release of the NBR and EPM composites, offering a new approach to imparting low flammability to elastomer materials.

## 1. Introduction

Over the past few decades, organic dyes based on azo chromophore have been widely applied as coloring agents in textile and polymer technologies. They owe their popularity primarily to the wide range of colors available and their relatively low price. Nevertheless, the very weak solvent resistance, low thermal stability, and weak photostability of some azo dyes restricts their application in more advanced polymeric materials. In order to overcome these problems and provide long-life polymer products, organic dyes are often combined with different inorganic supports such as silica, titanium dioxide, or montmorillonite [1,2,3,4].

Recently, a number of studies have focused on the preparation and characterization of organic-inorganic pigment composites [5,6,7,8,9,10]. Hybrid pigments appear to be an attractive alternative to conventional coloring agents due to the promise of combining coloring ability with the functionality of polymer fillers. The inorganic part (e.g., silica, gamma-alumina, titanium dioxide, or layered minerals) offers dye stabilization and simultaneously enables improvement of the functional properties (mechanical, thermal, and barrier) of polymer composites [11,12]. Organic-inorganic pigments have therefore attracted much attention from researchers interested in the design and fabrication of modern multifunctional coloring agents.

Aluminum-magnesium hydroxycarbonates are considered to be one of the most promising inorganic hosts, due to their large specific surface areas, ease of synthesis, good biocompatibility, and high anion exchange capacity [13,14,15]. The typical layered double hydroxide (LDH) molecular formulation can be described as [M^2+^_1−x_M^3+^_x_(OH)_2_]^x+^A^n−^_x/n_∙mH_2_O, where M^2+^ and M^3+^ represent divalent and trivalent cations, while A^n−^ corresponds to an interlayer ion such as CO_3_^2−^, NO_3_^−^, SO_4_^2−^, and so on [16,17,18,19,20].

Recently, intercalated/adsorbed aluminum-magnesium hydroxycarbonates were shown to be outstanding multifunctional hybrid pigments that could act as coloring agents and as fillers, imparting transparency, flame retardancy, a gas barrier, and high photo/UV and thermal stability to polymer composites. The literature suggests that the dispersal of hybrid pigments in elastomer medium could markedly improve barrier stability and flame retardancy, simultaneously ensuring high aesthetic qualities of the composites. Hybrid pigments based on LDH have been applied extensively in different thermoplastic polymers, such as polypropylene [21,22], polyethylene [23], polystyrene [24,25], and poly(vinyl alcohol) [26,27] to enhance mechanical, thermal, aging, and color characteristics. However, studies into the effects of organic-inorganic pigments on the performance of polymer composites have still been rather limited, especially regarding elastomeric matrices. Hajibeygi et al. [28] investigated the influence of organo-azo dye-modified aluminum-magnesium hydroxycarbonates on the thermal and optical properties of biobased semi-aromatic polyamide. Well-dispersed modified aluminum-magnesium hydroxycarbonates were found to be an efficient modifier for polyamide. 

In the current work, we propose new hybrid pigments obtained by the precipitation of carboxylic azo dyes onto aluminum-magnesium hydroxycarbonate with an Al:Mg weight ratio of 70:30. We applied these hybrid pigments as multifunctional colorants in acrylonitrile-butadiene (NBR) and ethylene-propylene (EPM) rubber composites. The effectiveness of the aluminum-magnesium hydroxycarbonate-based hybrid pigments for reducing the flammability of the studied composites was assessed by means of microcalorimetry Scanning electron microscopy (SEM) was used to investigate the morphology of the elastomer compounds. In addition, the impact of the pigment type on the color characteristics, curing behavior, crosslinking density, gas permeability, and static and dynamic mechanical properties of the cured elastomer composites was examined with various crosslinking systems.

## 2. Materials and Methods

### 2.1. Materials

**Figure 1:**
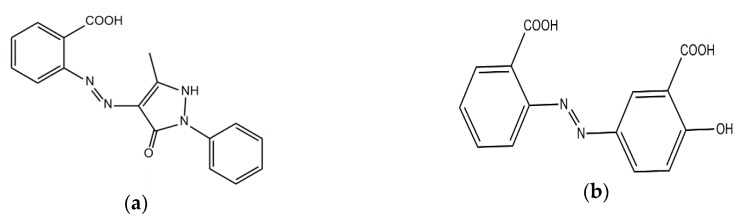
Chemical formulas of azo dyes: (**a**) Monocarboxylic azo dye (MCD); (**b**) dicarboxylic azo dye (DCD).

PURAL^®^ MG 30 aluminum-magnesium hydroxycarbonate (LH) (Al:Mg weight ratio 70:30, pore volume 0.5 mL/g) was purchased from Sasol, Hamburg, Germany). Modification of the LH was carried out using mono (MCD) and dicarboxylic (DCD) azo dyes (Figure 1). Azo dyes were synthesized according to the typical diazotization procedure, as described in the literature [29,30].

Acrylonitrile-butadiene rubber (NBR-EUROPRENE ® N2845 GRN, acrylonitrile content 28%, ML_1+4_ at 100 °C = 45) and ethylene-propylene copolymer (EPM-Dutral CO–054) were employed as elastomer matrices. The NBR compounds were crosslinked with a conventional sulfur-based system containing sulfur (Siarkopol, Tarnobrzeg, Poland), mercaptobenzothiazole (Siarkopol, Tarnobrzeg, Poland), zinc oxide (Huta Będzin, Będzin, Poland), and stearic acid (POCH S.A., Gliwice, Poland). The EPM compounds were cured using dicumyl peroxide (DCP, Sigma Aldrich, Schnelldorf, Germany) and 1,3,5-triallyl-1,3,5-triazine-2,4,6(1H,3H,5H)-trione (Sigma Aldrich, Schnelldorf, Germany) as co-agents of crosslinking.

### 2.2. Hybrid Pigment Synthesis

**Figure 2:**
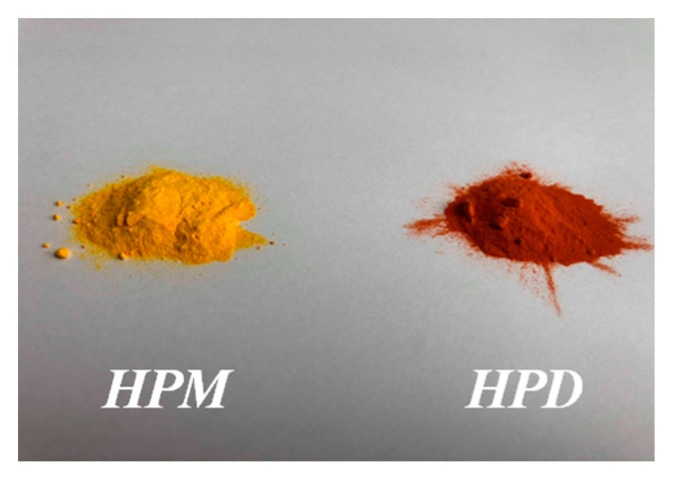
Digital photos of prepared hybrid pigments.

The hybrid pigments were synthesized according to the following procedure: 2 g of azo dye were dissolved in 200 cm^3^ of deionized water and 10 cm^3^ of ethyl alcohol. This mixture was then subjected to ultrasonication for 30 min. Next, the solution was heated to 80 °C, and aluminum-magnesium hydroxycarbonate (18 g) was added. The solution was stirred continuously for 3 h at 80 °C. Finally, the resultant pigment slurry was filtered and dried in an oven at 70 °C. As a result of these modifications, colorful hybrid pigment powders were obtained: LH modified with monocarboxylic dye (HPM) and LH modified with dicarboxylic dye (HPD) (Figure 2).

### 2.3. Composite Preparation

The hybrid pigments were used as multifunctional additives in NBR and EPM composites. Rubber blends were prepared in a two-step procedure. The rubber and organic-inorganic colorants were first homogenized in an internal mixer (Brabender Measuring Mixer N50) and then milled with a curing system in a laboratory mixing mill with the following parameters: Roll length (*L*) = 330 mm, diameter (*D*) = 140 mm, temperature 40 °C, friction = 1.1, rotation speed of the front roll (*V*_p_) = 20 rpm. The compositions of the rubber composites are listed in Table 1.

**Figure 3:**
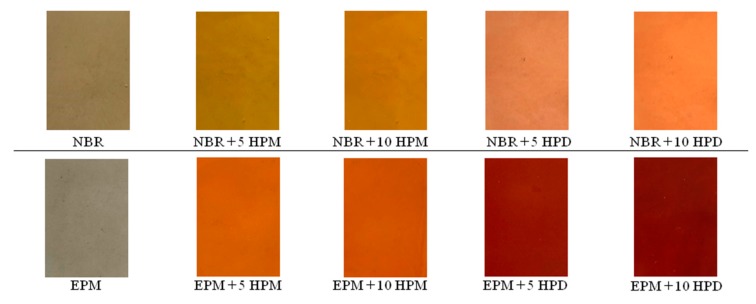
Digital photos of NBR and EPM composites containing hybrid pigments.

The composites were characterized by various colors, depending on the types of hybrid pigment and elastomer matrix applied (see Figure 3). As can be seen, as the content of pigment increased, the color of the composites became deeper and more vivid.

### 2.4. Studies of Hybrid Pigments

The hybrid pigments were subjected to a series of studies in order to characterize their structures. The solvent resistance of LH-based pigments was analyzed in accordance with the PN-C-04406 standard. The pigment powders were immersed in various organic solvents (ethanol, acetone, toluene, butyl acetate) for 24 h, after which the degree of decolorization was evaluated on a scale of 1–5. The thermal stability of the hybrid pigments was determined using thermogravimetric analysis (TGA). Thermal analysis was performed on a Q500 Thermogravimetric Analyzer (TA Instruments, Greifensee, Switzerand) with a heating rate of 10 °C/min across a temperature range of 25–600 °C, under an argon atmosphere. X-ray powder diffraction patterns were collected using an PANanalytical X’Pert Pro MPD diffractometer in Bragg–Brentano reflecting geometry with (CuK_α_) radiation from a sealed tube (Malvern Panalytical Ltd., Royston, UK). The apparatus operates in the range of 2*θ* = 2°–70°, with a step size of 0.0167. Elemental analyses of the carbon, hydrogen, and nitrogen elements in the hybrid pigment powders were carried out using a Vario EL III analyzer equipped with special adsorption columns and a thermal conductivity detector (TCD). The morphology of the hybrid pigments and their dispersion in the NBR and EPM composites were evaluated using a LEO 1530 Gemini scanning electron microscope (Zeiss/LEO, Oberkochen, Germany). Before the measurements, the composite films were broken in liquid nitrogen.

### 2.5. Kinetics of Curing

The curing kinetics of the elastomer compounds were studied in a moving die rheometer (MonTech MDR 300, Buchen, Germany) at 160 °C according to the ISO 3417 standard. Vulcanization was then performed using a standard electrically heated hydraulic press at a temperature of 160 °C, with a pressure of 15 MPa and according to the optimal curing time (τ_90_) determined in rheometric measurements.

### 2.6. Crossslink Density of Vulcanizates

The density of crosslinks in the vulcanizate network was determined based on rapid solvent-swelling measurements. The vulcanized samples were subjected to equilibrium swelling in toluene at room temperature. The crosslinking density values (*ѵ_e_*) were calculated using the Flory–Rehner equation [31],

(1) $${ѵ}_{e}=\frac{\ln\left(1-V_{r}\right)+V_{r}+\mu V_{r}^{2}}{V_{0}\left(V_{r}^{\frac{1}{3}}-\frac{V_{r}}{2}\right)},$$

where *V_r_* is the volume fraction of elastomer in swollen gel, and *V*_0_ is the molar volume of solvent [mol/cm^3^].

The polymer-solvent interaction parameters (μ_0_ and β) for each rubber were established experimentally and are presented in Table 2 [32,33].

### 2.7. Mechanical Strength of Composites

The tensile properties of the composites were evaluated using a universal testing machine (Zwick model 1435, Ulm, Germany), according to the ISO 37 standard. Tensile tests were performed at a crosshead speed of 500 mm/min at room temperature. Five specimens with a dumbbell shape for each series were applied to determine the average tensile strength.

### 2.8. Dynamic Mechanical Analysis (DMA)

Dynamic mechanical rheology measurements were conducted in tension mode using a DMA/SDTA861e analyzer (Mettler Toledo, New Castle, PA, USA). All dynamic tests were performed at a frequency of 1 Hz over a temperature range of −100 °C to 80 °C, with a heating rate of 2 °C/min and a strain amplitude of 10 μm. The glass transition temperatures of the elastomer composites were determined based on the maximum point from the tan δ = f(T) curves.

### 2.9. Air Permeability Measurement

To estimate the barrier properties of the elastomer composites, the manometric method was adopted, in accordance with the ASTM D1434 standard. Permeability measurements were carried out under atmospheric air conditions at room temperature. The barrier properties were defined based on the gas transmission rate (GTR) and the coefficient of gas permeability (P). These were estimated from the following equations:

(2) $$GTR=\frac{V_{C}}{R\cdot T\cdot P_{U}\cdot A}\cdot \frac{dp}{dt},$$

where *V*_C_ is the volume of the low-pressure chamber [l], R is the gas constant at 8.31×10^3^ [(l∙Pa)/(K∙mol)], *T* is temperature [K], *P_U_* is gas pressure in the high-pressure chamber [Pa], A is area permeation of gas through the sample [m^2^], and dp/dt is pressure change over time [Pa/s]. In addition,

(3) P=GTR·d,

where *d* is the thickness of the specimen [m].

### 2.10. Flammability of Composites (Microscale Combustion Calorimetry)

Each vulcanizate was examined in 2.5 mg samples using a microscale combustion calorimetry (MCC) microcalorimeter (Fire Testing Technology Limited). The temperature of the pyrolyser was 750 °C, and that of the combustor 900 °C. During the measurements, the following parameters were recorded: Maximum heat emission rate (HRR_max_), total heat emitted (THR), and heat capacity. The sample was heated using a linear temperature program. The volatile thermal degradation products were swept from the pyrolysis chamber by an inert gas and combined with excess oxygen in a tubular furnace at a temperature of 900 °C. This ensured the complete combustion (oxidation) of the fuel. The combustion products (CO_2_, H_2_O, and acid gases) were scrubbed from the gas stream. The transient heat release rate was calculated from the measured flow rate and oxygen concentration after correcting for flow dispersion. The maximum (peak) value of the MCC heat release rate, (HRR_MAX_) heat release rate was normalized for the initial sample mass. Heating rate is a material flammability parameter, measured in units of heat release capacity (J×g^−1^×K^−1^). This parameter depends only on the chemical composition of the sample and was proportional to the burning rate of the material in a fire.

## 3. Results and Discussion

### 3.1. Hybrid Pigment Characterization

**Figure 4:**
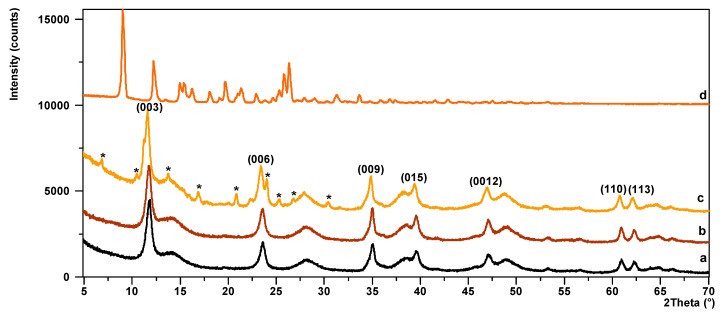
XRD powder diffraction patterns for (**a**) aluminum-magnesium hydroxycarbonate (LH), (**b**) HPD, (**c**) HPM, (**d**) MCD dye.

The XRD patterns of LH, HPD, and HPM are shown in Figure 4. The XRD pattern of the LH sample (Figure 4a) shows characteristic sharp and symmetrical peaks at low 2*θ* values, which were ascribed to diffractions by planes (003) and (006), corresponding to the basal spacing and its higher order diffractions. They are characteristic for crystalline hydrotalcite-like compounds, which form hexagonal lattices. On the slopes of the sharp peaks, small broad peaks can be observed, which may also have been due to unidentified crystalline impurities. After the addition of DCD or MCD dyes, the basal reflection (003) of the HPD and HPM pigments (Figure 4b,c) shifted slightly to a lower 2θ angle, but this did not change the interlayer distance significantly. The LH structure was retained. The most important difference observed in the diffraction patterns of HPM was the appearance of a new series of reflections that did not belong to either the LH or MCD dye. These reflections could have been caused by a new crystalline phase (marked with asterisks), which may have formed between the LH and MCD dye. The formation of the new hybrid materials was the result of interaction between the acidic groups of the dyes and ions present in the LH matrix, as will be discussed in the context of TG studies. The metal-azo dye interactions in such systems were already confirmed by time-of-flight secondary ion mass spectrometry (TOF-SIMS) analysis in our previous work [34]. The variation in the diffraction patterns of HPD and HPM dye was most likely related to differences in the structures of the azo chromophores. The modification of LH with MCD dye contributed to a greater formation of new crystals, probably due to the more complex structure of pyrazolone-azo dye in comparison to DCD.

**Figure 5:**
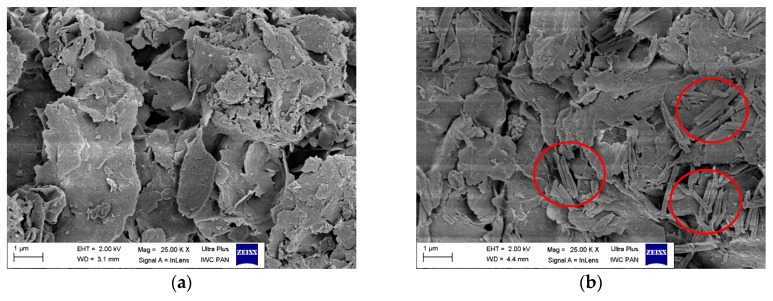

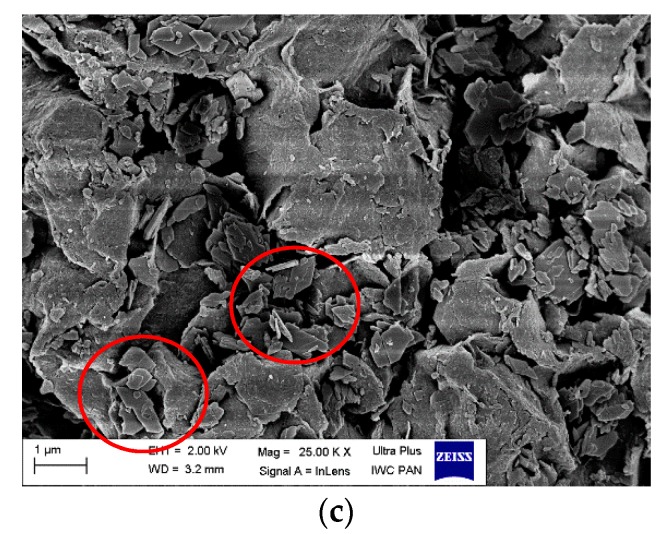
SEM micrographs of studied samples: (**a**) LH; (**b**) HPM; (**c**) HPD.

The morphologies of the LH host and synthesized hybrid pigments were inspected via scanning electron microscopy (SEM). The SEM images revealed the aluminum-magnesium hydroxycarbonate carrier to have an irregular layered structure, with dimensions ranging from 500 nm up to even a few µm (Figure 5a). As a result of modification with azo chromophores, some new crystals from another phase appeared on the LH surface (Figure 5b,c). Dye crystals of various shapes and dimensions were also observed, depending on the type of azo chromophore. The crystals of the monocarboxylic dye formed on the LH had a brick-like structure, while dicarboxylic dye formed plate-like crystals. Elemental analysis confirmed the presence of nitrogen in the hybrid pigments, which was characteristic for azo dyes, as the azo group −N=N− is part of their molecular structures (Table 3). This observation was in line with X-ray diffraction data and most likely resulted from the formation of new organic-inorganic hybrid structures. 

**Figure 6:**
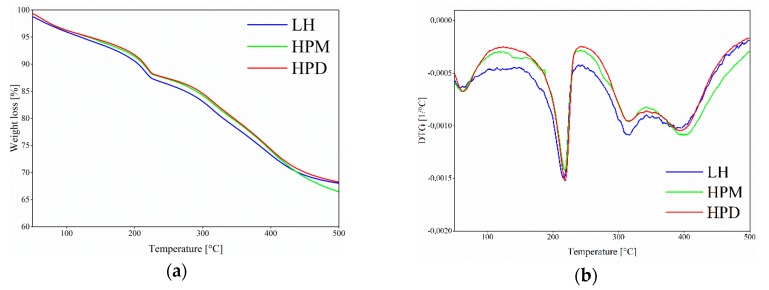
Thermal analysis of studied hybrid pigments: (**a**) thermogravimetric (TG) and (**b**) the derivative thermogravimetric (DTG) curves.

The modification of aluminum-magnesium hydroxycarbonate with azo chromophores resulted in hybrid pigments with different levels of thermal stability. Thermogravimetric differential analysis (TG-DTG) of the LH, HPM, and HPD revealed three weight-loss stages, as the temperature increased from 25 to 500 °C (Figure 6a,b). The first and second drops in weight between 25 and 250 °C could be ascribed to the removal of water molecules that had been absorbed into the surface and the interlayer space. The third weight loss between 300 and 500 °C corresponded with the dehydration of hydroxyl groups in the aluminum-magnesium hydroxycarbonate structure, as well as with the removal of carbonate anions (decarbonation) [35]. The TG results showed that the 5% weight loss temperatures for HPM (127 °C) and HPD (129 °C) were slightly shifted to higher temperatures than was the case with unmodified LH (119 °C). A similar effect was observed after dye modification in our previous work [36], in which we increased dye insolubility through the interaction of acidic groups of alizarin with particular ions present in aluminum-magnesium hydroxycarbonate (Al^3+^, Mg^2+^). The alizarin pigments demonstrated considerably lower weight loss above 300 °C, which was ascribed to the liberation of hydroxyl groups and carbonate ions from the layered mineral compared to unmodified LH. It was suggested that carbonate ions from the weak carbonate acid and water molecules were removed from the edge of the LH interlayer by acidic alizarin structures. The mechanism by which alizarin pigments were formed was similar to that for lake pigments, which are traditionally obtained by the reaction of dye molecules with metal salts [37]. A similar mechanism of metal-dye interaction is also suggested in the current study. It was concluded that slight changes in the thermal stability of the hybrid pigments were the result of interactions between magnesium or aluminum ions present in the inorganic carrier and carboxylic groups (–COO^−^) present in the dye molecules.

**Figure 7:**
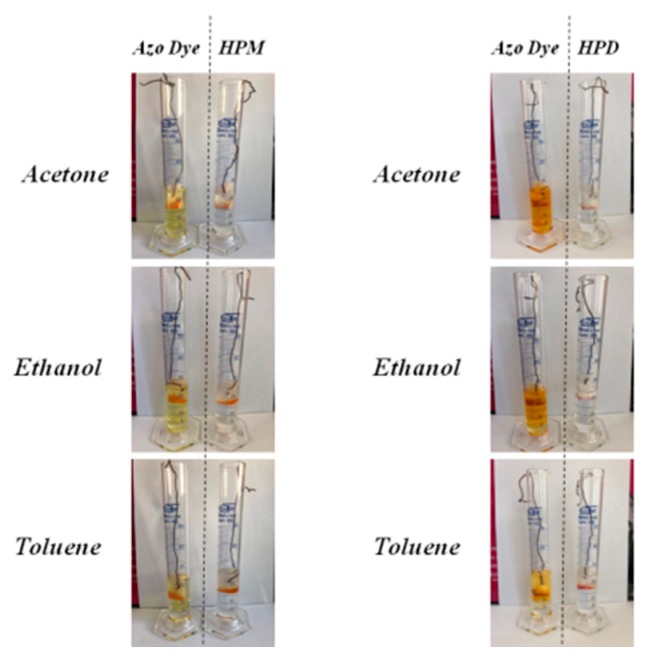
Digital photos of azo dye and hybrid pigment powders immersed in various organic solvents.

The uses of dyes are often limited due to their high solubility and tendency to migrate. One of the ways to overcome this disadvantage is the transformation of organic dyes into pigments, which are known for their insolubility and ability to create stable fine-grained dispersions in most applied mediums. The resistance of the organic-inorganic pigments and pure dye to discoloration was investigated by immersing studied powders in different solvents (Figure 7). The powder samples were placed in three different solvents (acetone, ethanol, and toluene), and the degree of discoloration was estimated after 24 h. Interestingly, the organic-inorganic pigments exhibited very strong resistance to dissolution in selected media (Figure 7). The obtained results could be attributed to the effective stabilization of dye molecules onto the LH surface. For both HPM and HPD samples, all of the solvents remained colorless, while unreacted chromophores demonstrated very intense color after 24 h of immersion. This indicated the successful modification of LH and transformation of azo chromophores into chemically stable hybrid pigments.

### 3.2. Kinetics of Curing

**Figure 8:**
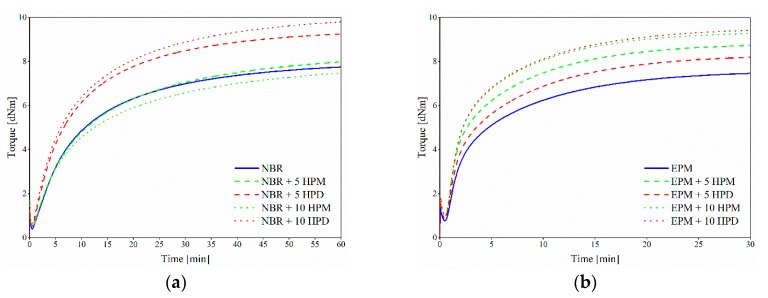
Curing curves for (**a**) NBR and (**b**) EPM compounds filled with hybrid pigments.

The influence of organic-inorganic pigments on the curing behavior of NBR and EPM compounds was estimated based on rheometric measurements. Figure 8 depicts curing graphs for NBR and EPM compounds filled with 5 phr and 10 phr of hybrid pigments. As can be seen, over time the blends containing pigments exhibited an abrupt increase in torque value compared to the reference. The cure properties of the elastomer composites are also summarized in Table 4. As expected, with increasing filler content, the minimum and maximum of the torque moment increased constantly for all compounds. This could be attributed to the hydrodynamic effect as well as to filler-filler and filler-polymer interactions [38]. The increase in ∆*M* with increasing pigment content was related to the presence of hybrid pigment particles in the matrix, which reduced the mobility of the macromolecules. The highest increment of torque was achieved for an NBR composite filled with 10 phr of HPD. A similar trend was observed with the EPM matrix. In contrast to those for HPD, no significant changes were observed in the rheometrical curves for blends with HPM in NBR systems. However, much higher curing efficiency was noticed when this filler was applied in the peroxide system (EPM matrix). The different effects of HPD and HPM additives on curing kinetics could be attributed to variations in the crystal structures of the hybrid pigments. It seems that the HPD pigment, which showed an XRD pattern similar to that of pristine LH, promoted a greater increase in torque values in both sulfur- and peroxide-based systems. It was concluded that the formation of the new crystal phase after modification with MCD affected the pigment-polymer interactions. As a consequence, HPM composites showed lower torque moment as well as increased optimum curing time in comparison to the HPD samples.

We also noted that the incorporation of hybrid pigments contributed to reduce both the scorch time and optimum curing time of the elastomer blends. The lower cure times and increased torque moments suggest that LH-based hybrid pigments could act as an additional accelerator in the curing process. Das et al. [39] also observed that both raw and modified layered double hydroxides could affect the crosslinking process in elastomer composites. 

### 3.3. Crosslink Density of Vulcanizates

**Figure 9:**
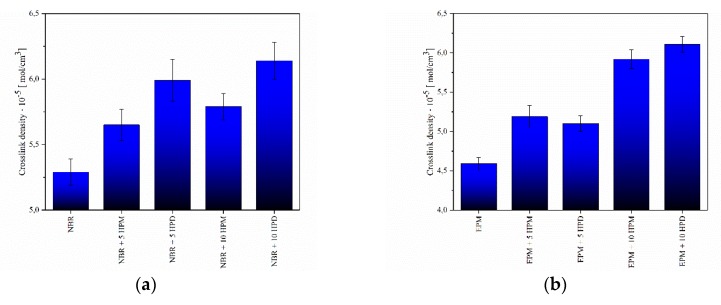
Crosslink density of (**a**) NBR and (**b**) EPM compounds filled with hybrid pigments.

In order to correlate the rheometric behavior and mechanical properties of the elastomer composites with their crosslink density, swelling experiments were performed in toluene. Ismail et al. [40] and Mouse et al. [41] reported that increased crosslink density values could be associated with reduced mobility in rubber, which makes it difficult for toluene to penetrate the material. Figure 9 presents the variations in crosslink density values of the studied composites. As can be seen, the crosslink density gradually increased with the loading of hybrid pigments. For both curing systems, the incorporation of 5 phr of hybrid pigment contributed to increasing the crosslink density by approximately 1 × 10^−5^ mol/cm^3^ in comparison to neat NBR or EPM. The higher crosslink density values could be explained by the homogeneous dispersion of hybrid pigments and their good interfacial interaction with polymer segments, with which the pigments formed a bound polymer that restricted solvent uptake from the surface to bulk. Furthermore, according to rheometric and crosslink studies, the presence of LH-based pigments may increase the efficiency of the curing process. The presence of unreacted metal oxides in the LH structure causes hybrid pigments to act as passive activators in the crosslinking process [39]. The results were in good agreement with those for rheometric measurements and could be attributed to the formation of more crosslinks in the vicinity of the hybrid pigments.

Moreover, the presence of 10 phr HPD pigment led to higher crosslink density values for NBR and EPM compared to samples containing HPM hybrid at the same concentrations. One of the reasons may be the fact that HPD powder, which showed a similar crystal structure to unmodified LH, may take part in and accelerate the curing process. Curing additives may be assumed to have better access to metal ions present in the HPD pigment than in the case of HPM, the outer surface of which is less covered by crystals originating from the chromophore.

### 3.4. Mechanical Strength of Composites

**Figure 10:**
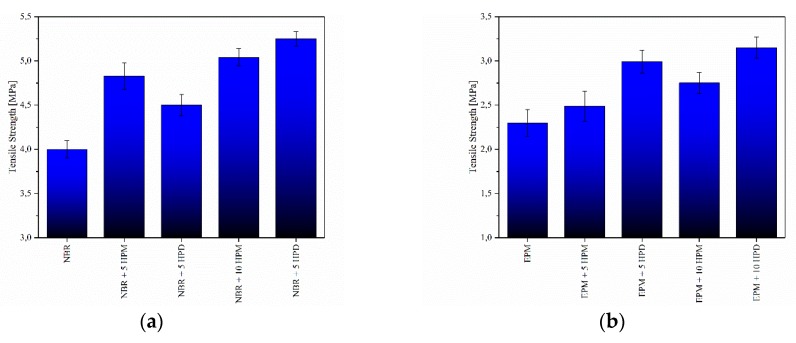
Tensile strength of (**a**) NBR and (**b**) EPM compounds filled with hybrid pigments.

The mechanical properties of the prepared composites were analyzed and comparted. Figure 10 shows the tensile strength values obtained for both unpigmented and pigmented elastomer compounds. In general, regardless of the matrix used, the presence of hybrid pigments caused mechanical reinforcement of the elastomers. In comparison to the control samples, the NBR- and EPM-filled composites exhibited enhanced tensile strength, which tends to increase in proportion with pigment loading. The highest reinforcement efficiency was recorded for the composites filled with 10 phr of HPD. The explanation for this may be the fact that the crystal structure of the HPD pigment was less affected by modification with organic chromophore in comparison to HPM (as evidenced by XRD). The improvements observed in tensile strength could be explained by the combined effects of good interphase interactions and the increased crosslink density of the elastomer composites.

### 3.5. Dynamic Mechanical Analysis

**Figure 11:**
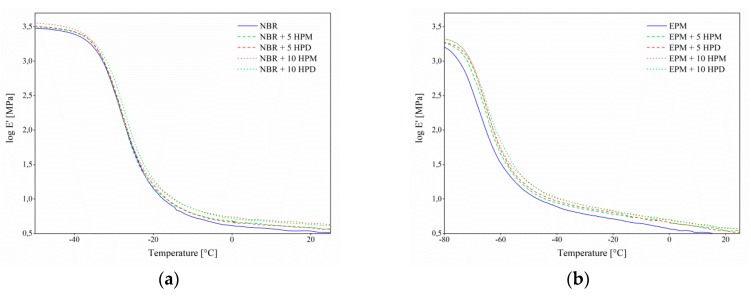
Storage modulus of (**a**) NBR and (**b**) EPM composites filled with hybrid pigments as a function of temperature.

**Figure 12:**
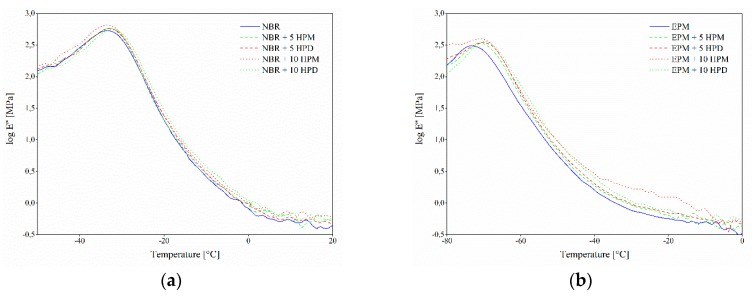
Loss modulus of (**a**) NBR and (**b**) EPM composites filled with hybrid pigments as a function of temperature.

Dynamic mechanical analysis was performed to investigate alterations in the molecular mobility of the polymer segments in the vicinity of the hybrid pigments. Figure 11 and Figure 12 show the variations in the storage (*E*′) and loss (*E*″) modulus for the studied elastomer composites. It can be noticed that the incorporation of HPM and HPD resulted in an increase in the storage and loss modulus of the NBR and EPM composites. However, the peroxide curing system (EPM compounds) was observed to have had a more significant influence on the modulus values. This improvement in the modulus could be attributed to the restricted flexibility of EPM molecular chains, due to strong interactions between the polymer chain and the pigment particles. The increased stiffness, resulting from enhanced crosslink density, may also have contributed to improve the mechanical properties of the EPM composites.

**Figure 13:**
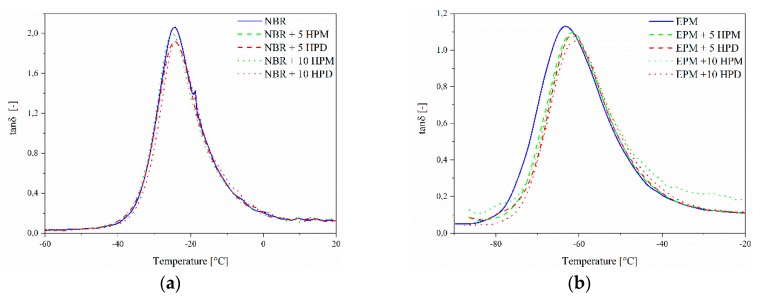
Tan δ of (**a**) NBR and (**b**) EPM composites filled with hybrid pigments as a function of temperature.

Figure 13 presents the tan δ versus temperature curves for the investigated composites. The tan δ peak is related to the polymer glass transition temperature (*T*_g_). In the glass transition region, the energy dissipation originates mainly from the segmental motion of the macromolecular chain of the rubber matrix [42]. Interestingly, the height of the tan δ peak was gradually reduced with increasing pigment loading in both the NBR and EPM matrixes, most probably due to the restricted mobility of the rubber chains. However, for EPM composites, the glass transition temperature values were noticed to shift about 2.0 °C toward higher temperatures with the addition of hybrid pigments, while for NBR no visible changes were observed. Since the *T*_g_ shift was not dependent on the pigment type and concentration, we suggest that the main reason for this effect may have been the nature of the curing system. It was already reported [43] that a sulfur-based curing system shows a tendency for adsorption onto the outer surfaces of the fillers. A similar behavior of curing additives in NBR composites was considered in the current study. Therefore, it seems that the slight increase in *T*_g_ of the EPM/hybrid pigments could be ascribed to better immobilization of the polymer and the physical blocking of molecular configurations as a result of the more effective polymer adsorption onto the filler surface.

### 3.6. Composite Morphology

**Figure 14:**
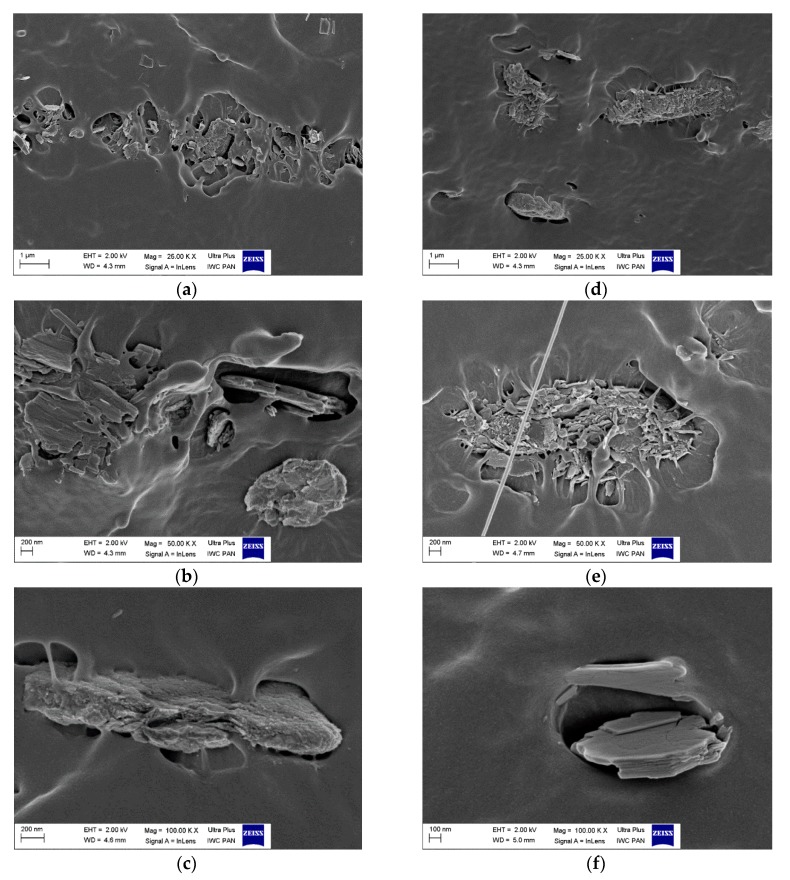
Scanning electron micrographs of (**a**–**c**) NBR and (**d**–**f**) EPM composites filled with 10 phr of hybrid pigment at different magnifications.

Morphological evaluation of the elastomer composites was carried out using scanning electron microscopy. Figure 14 shows SEM images of the fracture surface of NBR- and EPM-filled composites, respectively. As can be seen, the hybrid pigments after incorporation into NBR and EPM blends were still characterized by a layered structure of platelet-like shapes. They also exhibited satisfactory adhesion to the elastomer matrices. However, some small agglomerates were noticed, forming longitudinal paths or labyrinths in the composites (see Figure 14a). As can also be observed, some non-uniform distribution of the hybrid pigment particles appeared and may have affected the mechanical properties of the elastomer composites. The irregular dispersion of the LH-type pigments in the elastomer matrix may have been due to a few crucial factors, including high molecular weight, high viscosity, or the organic-inorganic incompatibility factor of these materials [16]. 

### 3.7. Barrier Properties

Generally, the presence of layered minerals can improve the gas barrier properties of elastomer composites due to the formation of paths hindering the diffusion of gas molecules within the polymer matrix. Therefore, we assumed that the hybrid pigments based on layered double hydroxides would strongly affect the air permeability of NBR and EPM composites. The gas transmission rate (GTR) and gas permeability coefficient (P) parameters were determined using the manometric method. The results are listed in Table 5.

The GTR and P parameters of the elastomer composites were found to have reduced considerably, in comparison to the pure NBR and EPM rubber. These decreases demonstrated the improved barrier properties of the composites. Air permeability also fell with increased hybrid pigment loading due to the higher crosslink density of the composites. The improvement in the barrier properties of the NBR and EPM composites could be attributed to two factors. The first was the geometric factor. The impermeable layers formed by the plate-shaped particles of hybrid pigment formed a labyrinthine pathway, which retarded the diffusion of gas molecules within the polymer matrix [44]. The second factor was related to the restricted motion of rubber chains by LH-based pigment particles, which reduced the diffusion of gas molecules [45].

### 3.8. Flammability of Composites

**Figure 15:**
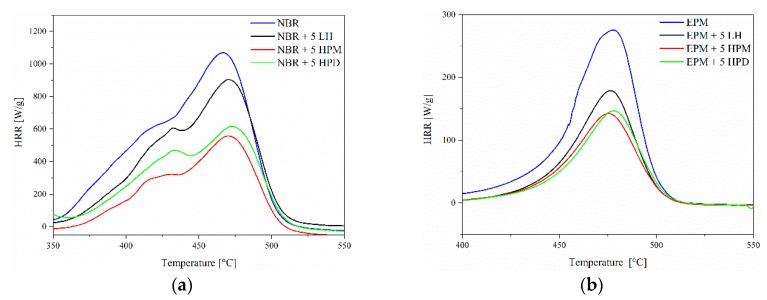
Heat release rate (HRR) curves of (**a**) NBR and (**b**) EPM composites, with a heating rate of 1 K/s.

Microscale combustion calorimetry (MCC) was used to predict the flame-retardant properties of the studied materials. Heat release rate (HRR) was one of the most important parameters monitored during this experiment and was used for evaluating compartment fire growth. Figure 15a,b shows the HRR plots for NBR and EPM composites containing LH, HPM, and HPD pigments measured by MCC, and the corresponding combustion data are displayed in Table 6. As can clearly be seen, HRR values were significantly lower in the case of elastomer composites with hybrid pigments at 5 phr and 10 phr concentrations. The HRR of NBR is 1091 W/g: It reduced to 893.5 W g^−1^ in NBR/5LH, 598.9 W g^−1^ in NBR/5HPM, and 612.3 W g^−1^ in NBR/5HPD, demonstrating significantly improved flame resistance (Table 6). The increase in flame resistance for EPM with hybrid pigments was less pronounced. The HRR value decreased from 271.9 W g^−1^ (EPM/5LH) to 145.5W g^−1^ (EPM/5HPM) and 149.3 W g^−1^ (EPM/5HPD). The reductions in the HRR peaks were also accompanied by decreases in the specific heat capacity (HRC) and total heat release (THR) values. The THR of NBR is 95.5 kJ g^−1^, while NBR containing LH and hybrid pigments showed lower THR values, with the peaks reduced to 66.3 kJ/g (NBR/5LH), 49.1 kJ/g (NBR/5HPM), and 47.6 kJ g^−1^ (NBR/5HPD). Even after taking into account the effect of dilution by the LH and hybrid colorants, the EPM composites showed enhanced flame resistance. The values for HRC were calculated as the sum of all peak HRR values. Pristine polymers gave the highest HRC, at 1059 J×g^−1^×K^−1^ (NBR) and 265 J×g^−1^×K^−1^ (EPM), while the NBR/5 HPD and EPM/5 HPD composites had markedly lower HRC values, of 599 J×g^−1^×K^−1^ and 146 J×g^−1^×K^−1^. The trend was therefore similar to that discussed previously in the case of HRR and THR. These results suggest that the incorporation of only small amounts of especially modified LH contributed to a considerable decrease in the THR values for the NBR- and EPM-based composites. Since the weight of the samples of composites used in measurements was very low (2.5 mg), we concluded that the observed increase in flame retardancy could not be explained by endothermic decomposition of the NBR and EPM composites. The improved flame retardancy of the polymers combined with LH and organic-inorganic pigments resulted most likely from the geometry and physicochemical structure of the aluminum-magnesium hydroxycarbonate host. 

The mechanism of flame retardancy in aluminum-magnesium hydroxycarbonate has been described previously [46]. It involves the formation of a refractory oxide residue on the surface of the polymer material and the subsequent release of water vapor and carbon dioxide during endothermic decomposition, which further facilitates the flame retardancy process. The plate-like shape of LH contributes to enhance the fire resistance of polymer materials because of the so-called “channel effect”, which itself is related to the limited diffusion of low-molecular products during thermal decomposition [47]. As a consequence, the heat and mass transfer between the gas and condensed phases is limited. The lower flammability of the investigated composites under the conditions of a real fire (e.g., a fire test using cone calorimetry) may also be connected with the limited diffusion of oxygen into the material, which reduces the yield of radical degradations during combustion and, as a consequence, increases its resistance to the effects of heat and flame. Permeability experiments clearly indicated that the addition of modified and unmodified aluminum-magnesium hydroxycarbonate decreased gas diffusion into both studied polymers (Table 5). Moreover, the HRR values of NBR/LH and EPM/LH composites were much higher in comparison to those of the NBR and EPM composites containing hybrid pigments. The greater effectiveness of the organic-inorganic colorants in terms of improving flame retardance of the polymer composites may have been related to the formation of new organic-inorganic structures and good pigment-polymer adhesion, as confirmed by SEM, which provided additional stabilization.

## 4. Conclusions

This paper reported on the preparation of new organic-inorganic pigments through the precipitation of azo dyes onto an aluminum-magnesium hydroxycarbonate host. Complex experimental techniques were applied to investigate the characteristics of the organic-inorganic pigments in various elastomer composites. The results showed that the hybrid pigments formed different crystal structures from those of raw LH, a sign of successful modification. The thermal and chemical stability of the elastomer composites also showed significant improvement. The pigments exhibited different behavior in sulfur and peroxide curing systems. For both elastomer systems, considerable increases in the torque moment, crosslink density values, tensile strength, and dynamic moduli were observed with increased hybrid pigment loading. These improvements may have been related to the good dispersion of the studied pigments (as evidenced by SEM) and strong filler-polymer interactions. Permeability studies further revealed improved barrier properties in comparison to unfilled NBR and EPM. The P and GTR parameters improved steadily with increasing pigment concentrations. The results of microscale combustion calorimetry showed that LH-based pigments could also decrease the flammability of the studied composites. The hybrid pigments also proved to be effective coloring agents. In conclusion, LH-based pigment fillers are a promising solution for obtaining advanced polymer composites with improved mechanical, thermal, and barrier properties, as well as aesthetic qualities.

## Figures and Tables

**Table 1:** Composition of acrylonitrile-butadiene (NBR) and ethylene-propylene (EPM) elastomer blends.

Materials	Content [phr ^1^]
NBR	100
Sulfur	2
Mercaptobenzothiazole	2
Zinc oxide	5
Stearic acid	1
EPM	100
Dicumyl peroxide	2
1,3,5-triallyl-1,3,5-triazine-2,4,6(1H,3H,5H)-trione	0.5
Hybrid pigment HPM (LH modified with MCD) Hybrid pigment HPD (LH modified with DCD)	0, 5, 10

^1^ phr: parts per hundred of rubber.

**Table 2:** Interaction coefficients between rubber network and toluene at 25 °C.

Rubber	μ_0_	β
NBR	0.381	0.671
EPM	0.501	0.273

**Table 3:** Elemental analysis of hybrid pigments.

Sample	%N	%C	%H
LH	-	1.55; 1.54; 1.52	3.20; 3.24; 3.25
HPM	2.15; 2.18; 2.17	9.22; 9.18; 9.25	3.37; 3.40; 3.41
HPD	0.77; 0.83; 0.80	7.51; 7.52; 7.50	3.40; 3.45; 3.48

**Table 4:** Curing parameters of NBR and EPM compounds.

Elastomer Compound	*M*_min_^1^ [dNm]	Δ*M* ^2^ [dNm]	τ_02_^3^ [min]	τ_09_^4^ [min]
NBR	0.41 ± 0.02	7.34 ± 0.01	3.57 ± 0.02	30.51 ± 0.02
NBR + 5 HPM	0.50 ± 0.01	7.49 ± 0.02	3.68 ± 0.02	33.61 ± 0.02
NBR + 10 HPM	0.51 ± 0.02	7.01 ± 0.02	3.67 ± 0.01	34.22 ± 0.01
NBR + 5 HPD	0.51 ± 0.01	8.74 ± 0.01	2.60 ± 0.01	27.84 ± 0.01
NBR + 10 HPD	0.63 ± 0.01	9.17 ± 0.03	2.50 ± 0.02	29.98 ± 0.02
EPM	0.77 ± 0.01	6.81 ± 0.01	1.54 ± 0.02	15.75 ± 0.01
EPM + 5 HPM	0.86 ± 0.02	7.87 ± 0.01	1.22 ± 0.02	13.23 ± 0.01
EPM+ 10 HPM	0.93 ± 0.02	8.34 ± 0.02	1.21 ± 0.03	12.59 ± 0.02
EPM + 5 HPD	0.86 ± 0.01	7.34 ± 0.01	1.36 ± 0.02	14.32 ± 0.01
EPM + 10 HPD	0.99 ± 0.03	8.43 ± 0.02	1.24 ± 0.03	13.09 ± 0.02

^1^ Minimum of torque moment; ^2^ increments of torque; ^3^ scorch time; ^4^ optimum curing time.

**Table 5:** Barrier properties of NBR and EPM composites filled with hybrid pigments.

**Parameter**	**NBR**	**5 HPM**	**10 HPM**	**5 HPD**	**10 HPD**
GTR ^1^	2.24×10^−7^	1.69×10^−7^	4.87×10^−8^	6.49×10^−8^	3.67×10^−8^
P ^2^	2.46×10^−10^	1.83×10^−10^	5.41×10^−11^	7.14×10^−11^	4.15×10^−11^
-	**EPM**	**5 HPM**	**10 HPM**	**5 HPD**	**10 HPD**
GTR	8.13×10^−8^	7.76×10^−8^	7.03×10^−8^	7.91×10^−8^	6.17×10^−8^
P	1.10×10^−10^	9.16×10^−11^	7.73×10^−11^	9.49×10^−11^	7.71×10^−11^

^1^ Gas transmission rate [mol/(m^2^∙s∙Pa)]; ^2^ gas permeability coefficient [mol∙m/(m^2^∙s∙Pa)].

**Table 6:** The flammability results recorded from the microscale combustion calorimetry (MCC) microcalorimeter.

Elastomer Compound	HRR_max_^1^ [W×g^−1^]	Total HR^2^ [kJ×g^−1^]	HR Capacity^3^ [J×g^−1^×K^−1^]
NBR	1091 ± 55	95.5 ± 4.8	1059 ± 64
NBR + 5 LH	894 ± 45	66.3 ± 2.7	865 ± 29
NBR + 10 LH	481 ± 29	68.0 ± 4.1	612 ± 31
NBR + 5 HPM	599 ± 24	49.1 ± 2.5	580 ± 23
NBR + 10 HPM	524 ± 31	39.2 ± 1.8	509 ± 25
NBR + 5 HPD	612 ± 31	47.6 ± 2.4	599 ± 30
NBR + 10 HPD	384 ± 12	46.5 ± 2.3	378 ± 15
EPM	275 ± 8	14.8 ± 0.7	268 ± 11
EPM + 5 LH	181 ± 7	7.3 ± 0.4	177 ± 9
EPM + 10 LH	112 ± 7	4.8 ± 0.2	110 ± 6
EPM + 5 HPM	146 ± 10	6.3 ± 0.3	142 ± 9
EPM+ 10 HPM	93 ± 5	4.1 ± 0.2	91 ± 5
EPM + 5 HPD	149 ± 6	6.2 ± 0.3	146 ± 7
EPM + 10 HPD	93 ± 6	3.9 ± 0.2	91 ± 4

^1^ Maximum heat release rate; ^2^ total heat release; ^3^ heat release capacity.
